# Liver sinusoidal cells eliminate blood-borne phage K1F

**DOI:** 10.1128/msphere.00702-23

**Published:** 2024-02-28

**Authors:** Javier Sánchez Romano, Jaione Simón-Santamaría, Peter McCourt, Bård Smedsrød, Kim Erlend Mortensen, Antonia P. Sagona, Karen Kristine Sørensen, Anett Kristin Larsen

**Affiliations:** 1 Department of Medical Biology, UiT The Arctic University of Norway, Tromsø, Norway; 2 Gastrointestinal Surgery Unit, University Hospital of North Norway, Tromsø, Norway; 3 School of Life Sciences, University of Warwick, Coventry, United Kingdom; University of Michigan, Ann Arbor, Michigan, USA

**Keywords:** bacteriophage, blood clearance, Kupffer cells, liver sinusoidal endothelial cells, organ distribution, scavenger cells

## Abstract

Phage treatment has regained attention due to an increase in multiresistant bacteria. For phage therapy to be successful, phages must reach their target bacteria in sufficiently high numbers. Blood-borne phages are believed to be captured by macrophages in the liver and spleen. Since liver sinusoids also consist of specialized scavenger liver sinusoidal endothelial cells (LSECs) and Kupffer cells (KCs), this study investigated the contribution of both cell types in the elimination of *Escherichia coli* phage K1Fg10b::gfp (K1F^gfp^) in mice. Circulatory half-life, organ, and hepatocellular distribution of K1F^gfp^ were determined following intravenous administration. Internalization of K1F^gfp^ and effects of phage opsonization on uptake were explored using primary mouse and human LSEC and KC cultures. When inoculated with 10^7^ virions, >95% of the total K1F^gfp^ load was eliminated from the blood within 20 min, and 94% of the total retrieved K1F^gfp^ was localized to the liver. Higher doses resulted in slower elimination, possibly reflecting temporary saturation of liver scavenging capacity. Phage DNA was detected in both cell types, with a KC:LSEC ratio of 12:1 per population following cell isolation. Opsonization with plasma proteins increased time-dependent cellular uptake in both LSECs and KCs *in vitro*. Internalized phages were rapidly transported along the endocytic pathway to lysosomal compartments. Reduced viability of intracellular K1F^gfp^ corroborated inactivation following endocytosis. This study is the first to identify phage distribution in the liver at the hepatocellular level, confirming clearance of K1F^gfp^ performed mostly by KCs with a significant uptake also in LSECs.

**IMPORTANCE:**

Faced with the increasing amounts of bacteria with multidrug antimicrobial resistance, phage therapy has regained attention as a possible treatment option. The phage field has recently experienced an emergence in commercial interest as research has identified new and more efficient ways of identifying and matching phages against resistant superbugs. Currently, phages are unapproved drugs in most parts of the world. For phages to reach broad clinical use, they must be shown to be clinically safe and useful. The results presented herein contribute to increased knowledge about the pharmacokinetics of the T7-like phage K1F in the mammalian system. The cell types of the liver that are responsible for rapid phage blood clearance are identified. Our results highlight the need for more research about appropriate dose regimens when phage therapy is delivered intravenously and advise essential knowledge about cell systems that should be investigated further for detailed phage pharmacodynamics.

## INTRODUCTION

Faced with the worldwide increase in infections with antibiotic multiresistant bacteria, interest in phage treatment to control bacterial infections has regained attention. Although having a long history, with their discovery more than a century ago [reviewed in reference (1)], routine clinical use of phages as antibacterial therapy has faced obstacles and is not yet a mainstream approach. For phage therapy to succeed, phages must reach their susceptible target bacteria in sufficiently high numbers. There are several factors that may pose obstacles to successful phage and bacteria encounter, including phage’s ability to penetrate mammalian host tissues and travel through body compartments (absorption and distribution), as well as innate and acquired immune reactions and other factors in the host receiving phage treatment that contributes to phage inactivation (metabolism) and excretion (2). Phage therapy can be delivered by several routes [oral or parenteral (topical, inhaled, or injected)] depending on the type of infection to be treated. The most efficient, and often strictly necessary route of delivery when treating systemic infections, is intravenous administration. Systematic studies of phage pharmacokinetics and pharmacodynamics in mammals receiving phage therapy are limited, and few reports exist about the cells and mechanisms involved in phage blood clearance (3). Internalization of phages by our body cells is postulated as a major process determining pharmacokinetics, but it has been studied mostly in immortalized cell lines, which are often very different from normal cells in the body (4). Furthermore, phages are highly diverse entities and differ in size, shape, surface structure, and charge, which affect their uptake in cell lines (3, 5).

Early studies in the 1950s noted that the concentration of a *Staphylococcus* phage, strain 53, in the blood of rabbits immediately after injection (2.5  min) was 100-fold lower than the hypothetical titer calculated from the phage dose and its dilution in total blood volume (6). Other studies confirmed that measured phage concentrations in blood were much lower than expected, both according to theoretical dilution in total blood volume also according to hypothetical dilution in total body volume [reviewed in reference (3)]. This effect is believed to be due to the capture of phages by cells belonging to the reticuloendothelial system (RES), defined by Aschoff (7). Although Aschoff did not claim that the RES was made up of macrophages, over the years following his RES proposal, the understanding that the RES was synonymous with macrophages was gradually accepted. After influential macrophage researchers advised that the term RES should be replaced entirely by the term mononuclear phagocyte system (MPS) (8), few, if any, contended the notion that RES = MPS. As important RES organs, the liver and spleen have been reported to accumulate phages delivered systemically (9, 10). Following rapid removal from the circulation in mice, T4 bacteriophages were concentrated in the liver 12 times the level in the spleen (10), an effect assumed to be mediated by the resident liver macrophages, the Kupffer cells (KCs). Of note, these studies, published about 50 years ago and constituting the current basis of phage distribution knowledge, were undertaken before the liver RES was redefined to comprise a dual scavenger cell system, representing KCs and liver sinusoidal endothelial cells (LSECs) (11–13).

The liver is the largest internal organ, with important functions both in metabolism and in host defense. It contains the single largest number of resident macrophages (i.e., KCs) and the highest concentration of natural killer cells and natural killer T-cells in the body. In addition, the LSECs, which make up the entire lining of the hepatic sinusoids, are highly active scavenger cells and critical for maintaining immune homeostasis within the liver (13–15). As opposed to KCs, which are professional phagocytes and responsible for the clearance of bacteria and dead cells, LSECs are essentially non-phagocytic. They operate largely via clathrin-mediated endocytosis (13, 14) and are responsible for the removal of many proinflammatory macromolecules, nanosized particles, and colloids, including several viruses from the circulation (14, 16, 17). Together, these two cell types constitute the most powerful scavenger cell system in the body.

Most reports on hepatic clearance of blood-borne viruses focused on the role of phagocytosis in KCs, which are frequently and mistakenly understood as the only player in the liver RES (12, 13, 18). However, viruses are colloidal nanoparticles, and a variety of viruses rapidly disappear from the circulation, e.g., polyomavirus BK, and BK and JC polyomavirus (PyV)-like particles (17, 19), simian immunodeficiency virus (20, 21), HIV-like particles (22), or wild-type and recombinant human adenovirus type 5 (16, 23) with the liver being the main uptake organ and the LSEC being singled-out as the cell type mainly responsible for the rapid blood clearance (16, 17, 22).

**Fig 1:**
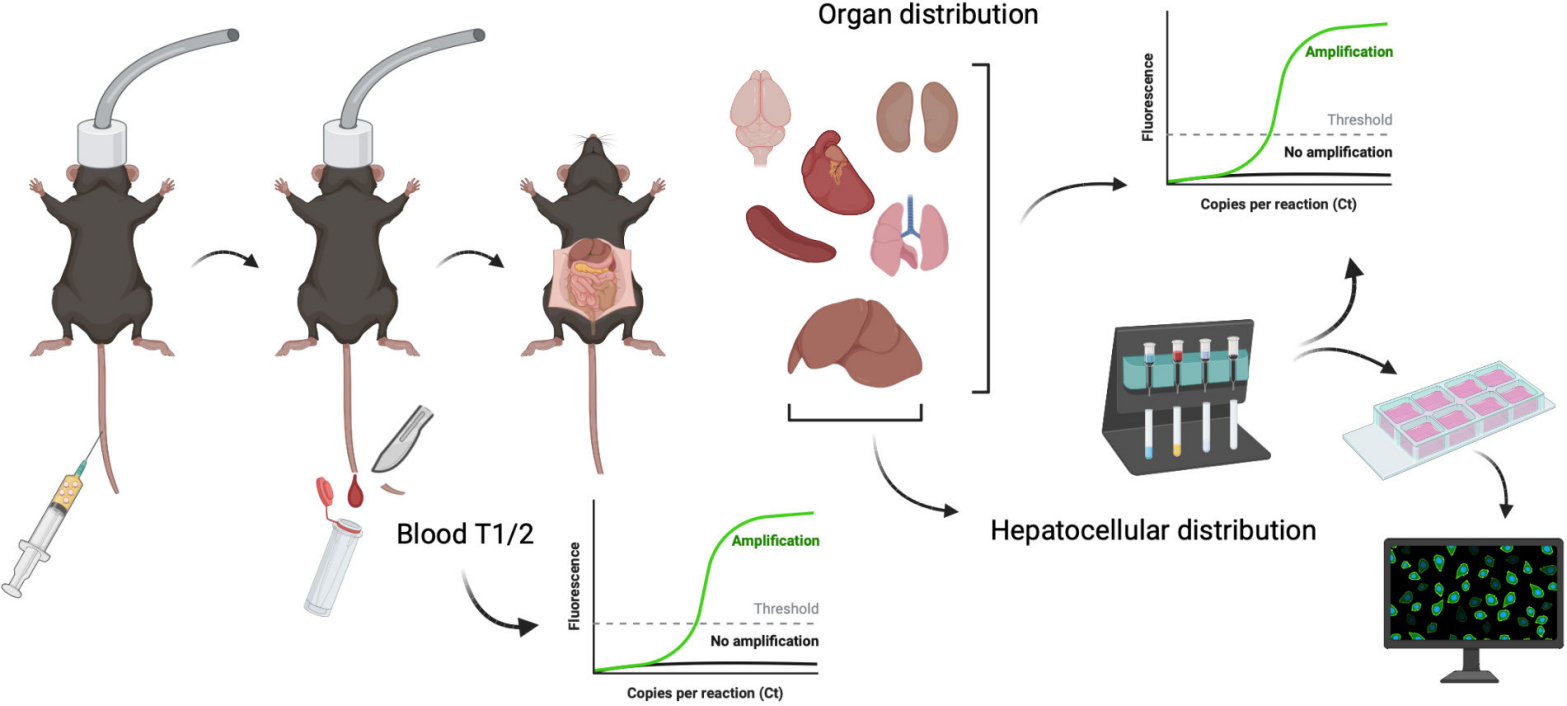
Brief outline of the experimental setup for the *in vivo* study. Created with BioRender.com.

Our leading hypothesis is that due to the extraordinarily high endocytic capacity of LSECs, many medium-sized and small phages reaching the liver can be internalized by these cells and are then efficiently degraded. We recently reported that T4 phages are endocytosed and degraded by primary rat LSECs *in vitro* (24). In the present study, we aimed to investigate the involvement of different liver scavenger cells in the rapid elimination of phages from circulation. To this end, blood clearance, organ distribution, and intracellular transport of the small T7-like phage K1F (25) were investigated in the C57BL/6JRj mouse model (Fig. 1). In order to evaluate the contribution of LSECs in blood clearance of phages, it was important to investigate the fate of the circulating phages within minutes after intravenous administration, as uptake in the liver RES and, in particular, LSECs is a rapid process (14).

## RESULTS

### Rapid removal of bacteriophage K1F^gfp^ from blood

**Fig 2:**
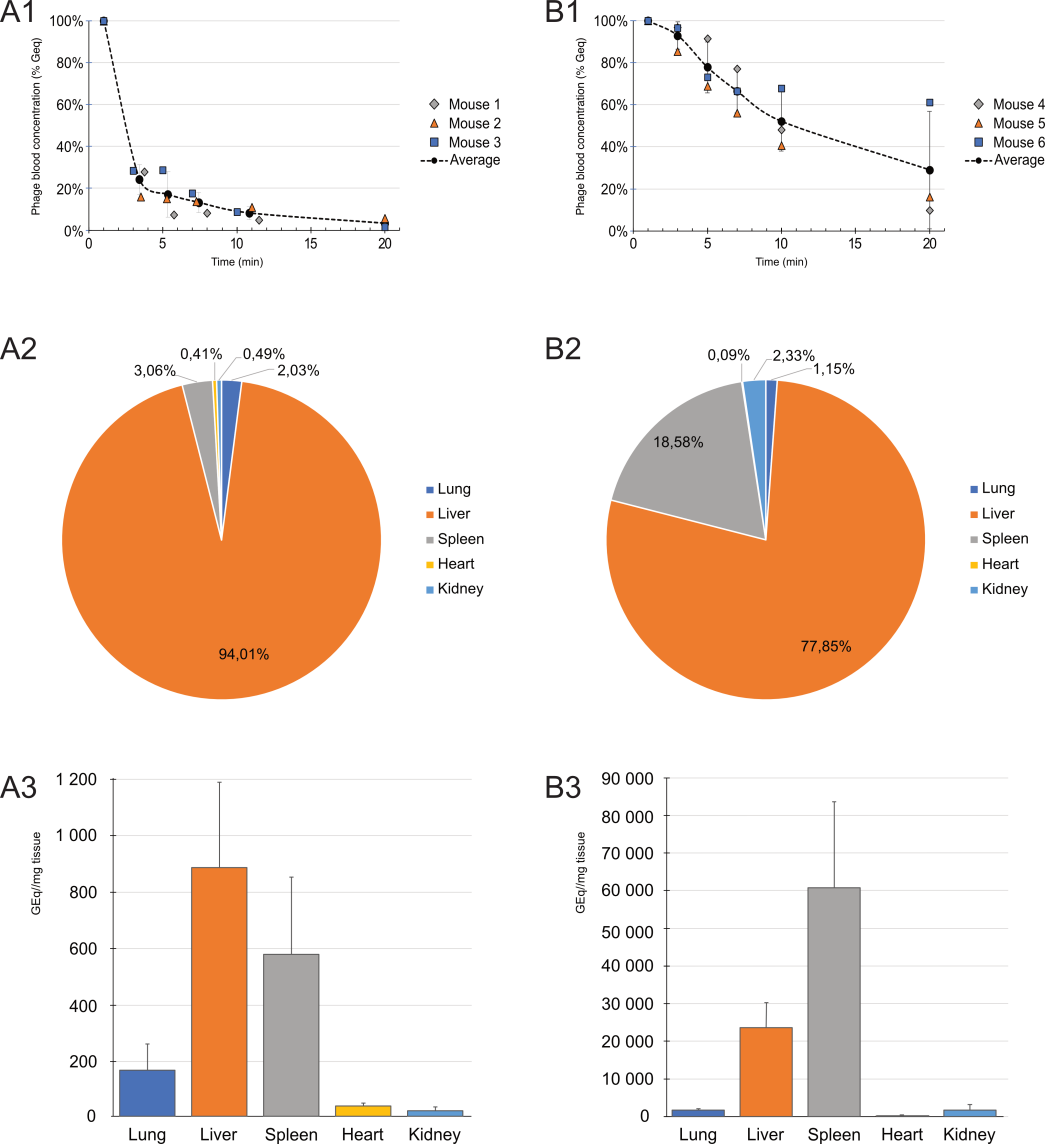
Blood clearance and organ distribution of phage K1F^gfp^ in C57BL/6JRj mice receiving either 10^7^ (**A**) or 6.45 × 10^9^ (**B**) genome equivalents (GEq) intravenously. Mice (*n* = 3) were injected with phage K1F^gfp^ in the tail vein, and blood clearance in the first 20 min following challenge was determined. Graphs show the percentage of initial dose K1F^gfp^ detected at 1 min post injection. Rapid clearance of phage particles was observed in both groups. Animals receiving 10^7^ (**A1**) almost reached complete clearance, while animals receiving 6.45 × 10^9^ (**B1**) cleared almost 3/4 of the total dose given. Per organ, the liver was associated with the highest number of phage DNA detected. In the lower dose (**A2**), the majority was eliminated by the liver, with only small amounts detected in the spleen, lung, heart, and kidney. At the higher dose (**B2**), liver was found to eliminate the largest amount, with some contribution from the spleen. Calculated per milligram tissue, the liver had the higher concentration of GEq/mg in the mice receiving the lower dose [10^7^ (**A3**)], while the spleen was associated with most phage DNA in the higher dose [6.45 × 10^9^ (**B3**)]. Bars in (**A3**) and (**B3**) represent mean with error bars representing the SEM.

Measurement of phage DNA in blood samples collected from 1 to 20 min after intravenous administration revealed that the blood clearance of K1F^gfp^ was dependent on inoculated dose. Following injection with 10^7^ K1F^gfp^ particles, 71.4%–84.1% of the particles were eliminated from blood within 3 min, and only 1.4%–5.6% of the dose remained in the circulation after 20 min (Fig. 2A1). Injection with 6.45 × 10^9^ K1F^gfp^ particles resulted in a slower clearance rate and more variation between animals (Fig. 2B1), with 9.7%–61.1% of the original retrieved dose at 1 min still in circulation after 20 min.

### Circulating K1F^gfp^ is eliminated from blood mainly by the liver

Quantification of viral genomes in selected tissues (liver, spleen, lung, heart, kidneys, and brain) also varied depending on the administered dose of K1F^gfp^. Figure 2 shows that the liver is instrumental in early blood clearance. Quantitative real-time PCR (qPCR) results from organ samples collected from mice receiving the lowest dose showed that 94.0% (*n* = 3, SD = 5.5%) of the total retrieved amount of K1F^gfp^ was localized to the liver (Fig. 2A2). Mean K1F^gfp^ level in the liver was 1.11 × 10^6^ (*n* = 3, SD = 6.65 × 10^5^) genome equivalents (GEq)/organ. Viral genomes were also detected in spleen and lung, each receiving 3.1% and 2.0% of the total K1F^gfp^ load, respectively, in total 3.60 × 10^4^ (*n* = 3, SD = 2.97 × 10^4^) and 2.41 × 10^4^ (*n* = 3, SD = 1.39 × 10^4^) GEq/organ. Low amounts of K1F^gfp^ were detected in the heart (4.80 × 10^3^ GEq/organ; *n* = 3, SD = 2.35 × 10^3^) and kidneys (5.81 × 10^3^ GEq/organ; *n* = 3, SD = 7.10 × 10^3^), corresponding to 0.5% and 0.4% of the retrieved amount of phage DNA, respectively. Results from organ samples collected from mice receiving the highest dose of K1F^gfp^ showed that 77.9% (*n* = 3, SD = 1.7%) of the total retrieved amount of K1F^gfp^ was localized to the liver, while 18.6% (*n* = 3, SD = 7.7%) was in spleen (Fig. 2B2). Mean K1F^gfp^ levels in liver and spleen were 2.24 × 10^7^ (*n* = 3, SD = 9.21 × 10^6^) and 5.34 × 10^6^ (*n* = 3, SD = 3.70 × 10^6^) GEq/organ, respectively. Viral GEq were also detected in lung (3.30 × 10^5^; *n* = 3, SD = 9.60 × 10^4^), heart (2.67 × 10^4^; *n* = 3, SD = 2.68 × 10^4^), and kidneys (6.69 × 10^5^; *n* = 3; SD = 9.68 × 10^5^). Calculated per milligram tissue, the liver was associated with highest amount of phage DNA in the low dose, while the spleen had more phage DNA/mg tissue in the high dose (Fig. 2A3 and B3). Total amounts of viral GEq/mg tissue weight and total organ weights can be found in Table S1.

### LSECs and KCs are both involved in the elimination of K1F^gfp^ from the circulation

Hepatocellular distribution of phage K1F^gfp^ following rapid blood clearance was investigated by assessing phage DNA in isolated KCs and LSECs 10 min post administration of 10^8^ GEq intravenously. Analyses of the different cell fractions showed that K1F^gfp^ was associated with both KCs and LSECs. Following adjustment for cell purity and extrapolation to the number of KCs and LSECs/liver, the content of phage DNA was found to be 12 times higher (±9.26×, *n* = 3) in the KC population than in the LSEC population (Table 1).

**TABLE 1:** Hepatocellular distribution of phage K1F^gfp^ from cell fractions isolated from C57BL/6JRj male mice (*n* = 3)^*c*^

Sample ID		Cells isolated	Purity (%)	GEq/100,000 cells	Total GEq/sample	Cells isolated/liver^*b*^	GEq/cell population	Ratio GEq^KC^/GEq^LSEC^
K1F007	LSEC	2.50 × 10^6^	97.61	8,90 × 10^2*a*^	2.28 × 10^4*a*^	7.39 × 10^6^	6.90 × 10^4*a*^	22.89
	KC	4.00 × 10^5^	96.08	1.32 × 10^5^	5.27 × 10^5^	5.74 × 10^5^	1.58 × 10^6^	
K1F008	LSEC	1.35 × 10^6^	97.32	3.91 × 10^3*a*^	5.43 × 10^4^*^a^*	3.98 × 10^6^	1.65 × 10^5*a*^	7.00
	KC	2.50 × 10^5^	96.08	1.53 × 10^5^	3.84 × 10^5^	3.59 × 10^5^	1.15 × 10^6^	
K1F009	LSEC	5.00 × 10^6^	98.36	3.36 × 10^3*a*^	1.71 × 10^5*a*^	1.49 × 10^7^	5.17 × 10^5*a*^	6.71
	KC	7.00 × 10^5^	98.77	1.65 × 10^5^	1.16 × 10^6^	1.03 × 10^6^	3.47 × 10^6^	

^*a*^ GEq has been adjusted to account for contamination of KCs in the sample.

^*b*^ Extrapolated from cell numbers received from 1/3 (LSECs) and 2/3 (KCs) of the total non-parenchymal cells isolated/liver.

^*c*^ Mouse LSECs were isolated 10 min after phage injection by positive selection using CD146 conjugated magnetic microbeads, while KCs were isolated by positive selection using a combination of F4/80 conjugated beads and CD11b conjugated beads. Results are shown in GEq per 100,000 cells and extrapolated to the total amount of each liver cell population. The ratio GEq^KC^/GEq^LSEC^ shows how many virions are present in the isolated KC fraction compared to the LSEC fraction.

### Internalization of K1F^gfp^ in LSECs and KCs is augmented by plasma protein opsonization

**Fig 3:**
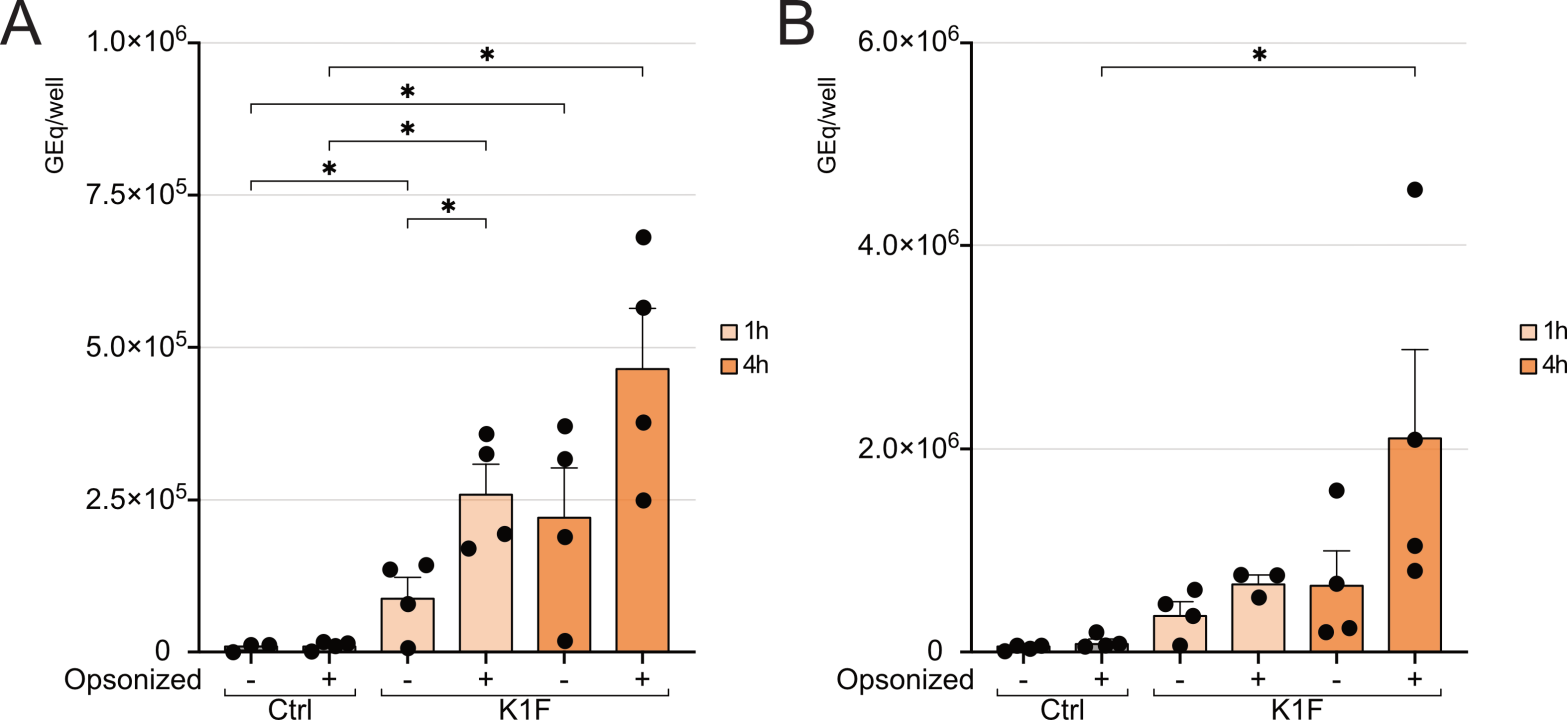
Uptake of native (−) or plasma protein-opsonized (+) phage K1F^gfp^ in primary mouse LSECs (**A**) and KCs (**B**) *in vitro*. Cells were challenged with 0.5 × 10^8^ PFU in a total volume of 150 µL/well for 1 or 4 h. Internalization of both native and opsonized phages increased with time in both cell types. Bars show the mean of samples from four mice run in two separate assays for LSEC, with individual values from each mouse shown as dots. For KCs, the yield is lower, and cells from two to four livers were pooled in each assay, representing one biological replicate. The bars represent the mean of samples from three or four assays, with individual values shown as dots. Error bars represent SEM. Asterisks (*) indicate significantly different from respective control or different from native phages at similar timepoint, *P* < 0.05.

To determine if the rate of phage internalization was affected by plasma protein opsonization, primary LSEC and KC cultures were challenged with 0.5 × 10^8^ plaque-forming units (PFU) of native or plasma opsonized K1F^gfp^/2 × 10^5^ cells for 1 or 4 h before cell-associated phage DNA (GEq) was quantified (Fig. 3). Cell-associated phage numbers were normalized by subtracting the number of phages detected in the medium in the last washing step. In addition, control samples were challenged with phages for 5 min at room temperature (RT) before wells were washed and incubated for 1 h, representing unspecific binding of phage particles. Figure 3 shows that phage K1F^gfp^ was effectively internalized in both LSECs and KCs. Challenging with native phages for 1 h yielded mean uptake of 9.13 × 10^4^ GEq/well (*n* = 4, SD = 6.30 × 10^4^) in LSECs (Fig. 3A) and 3.80 × 10^5^ GEq/well (*n* = 4, SD = 2.35 × 10^5^) in KCs (Fig. 3B). Levels of internalized native phage particles showed a steady increase over time, with uptake rising from 1 to 4 h by factors of 2.45 and 1.78 in LSECs [2.24 × 10^5^ GEq/well (*n* = 4, SD = 1.57 × 10^5^)] and KCs [6.75 × 10^5^ GEq/well (*n* = 4, SD = 6.47 × 10^5^)].

Internalization of opsonized phages (pretreated for 30 min with mouse plasma) was significantly higher already at 1 h for LSECs with mean GEq/well of 2.62 × 10^5^ (*n* = 4, SD = 9.36 × 10^4^), i.e., more than double the amount compared to uptake of native phages at 1 h challenge. At 4 h, the uptake of opsonized phages had increased by a factor of 2.1 compared to native phages at the same timepoint [mean 4.68 × 10^5^ GEq/well (*n* = 4, SD = 1.92 × 10^5^); Fig. 3A]. KCs internalized opsonized phages 1.8 times more at 1 h [mean 6.88 × 10^5^ GEq/well (*n* = 3, SD = 1.23 × 10^5^)] and 3.7 times more at 4 h [mean 2.41 × 10^6^ GEq/well (*n* = 4, SD = 2.30 × 10^6^); Fig. 3B] compared to uptake of native phages.

### Internalized phage K1F^gfp^ is distributed to the endosomal compartment

**Fig 4:**
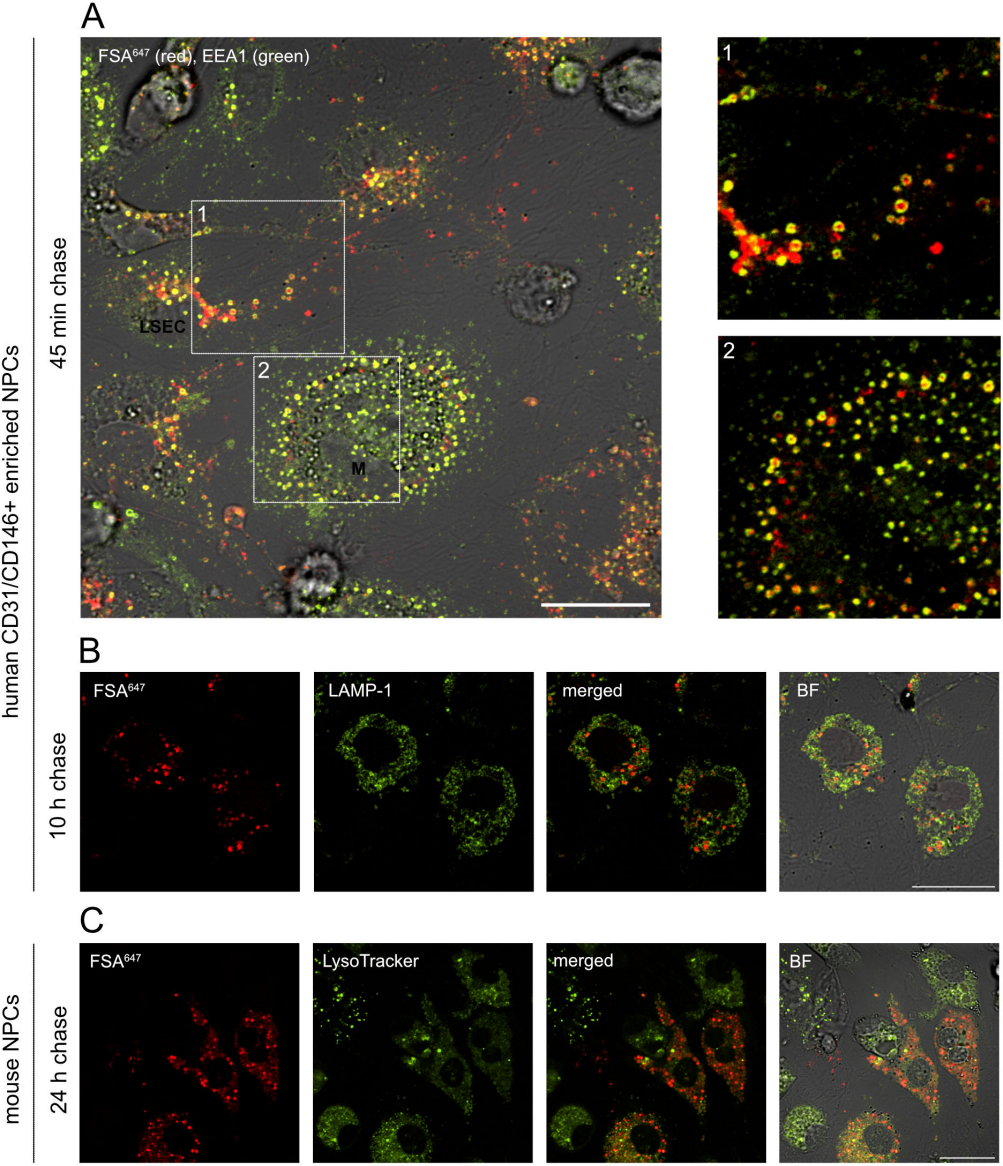
Human primary sinusoidal cell cultures enriched in LSECs and mouse primary NPC cultures rich in LSECs and KCs were challenged with 10 µg/mL FSA^647^ for 15 min and pulse chased for indicated times. After 45 min, FSA^647^ (red) was localized in EEA1-positive vacuoles (green) in human LSECs (**A1**) and liver-associated macrophages (**A2**). After 10 h, vacuoles containing FSA^647^ were localized inside or close to perinuclear ring-like LAMP-1 positive structures (**B**). By 24 h, the FSA^647^-positive structures were completely overlapping structures labeled with LysoTracker (**C**). Fluorophores captured in the 550–590 excitation range (EEA1, LAMP-1, and LysoTracker Red DND99) are depicted in green for better visualization in the images. Scale bar = 20 µm.

**Fig 5:**
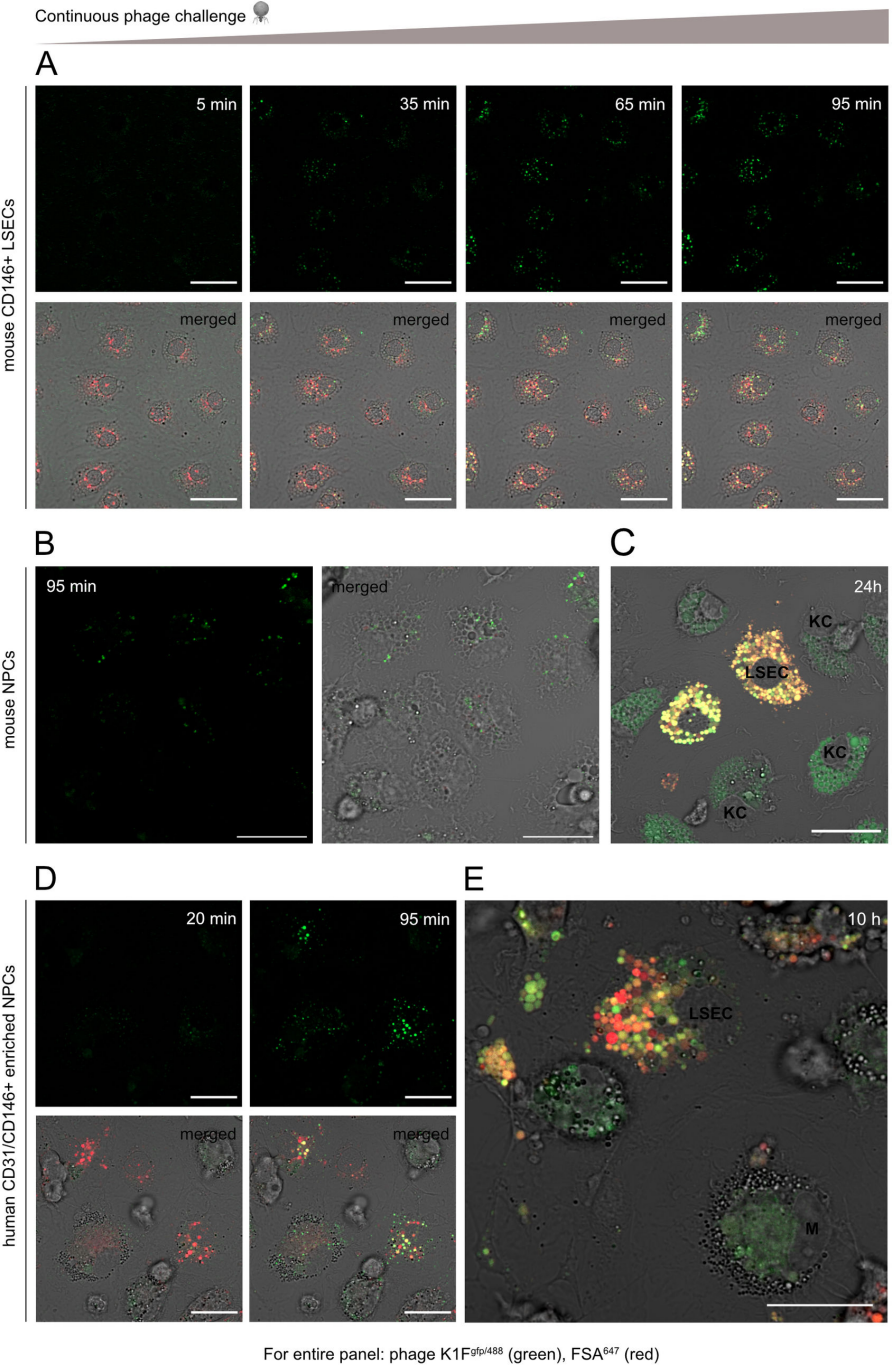
Live cell imaging of continuous phage challenge in primary LSEC and NPC cultures. Cells were pre-treated with the FSA^647^ and challenged with 2 × 10^9^ PFU of Alexa Fluor 488-labeled K1F^gfp^ (K1F^gfp/488^) in a total volume of 500 µL/dish. Images were captured using a Zeiss LSM800 confocal microscope. Phages (green) were rapidly internalized in mouse (**A**) and human (**D**) LSECs and colocalization with FSA^647^ (red) increased with time. Phage uptake was also evident in mouse KCs (**B**). After 24 h, almost all K1F^gfp/488^-containing vesicles were overlapping with FSA^647^-positive perinuclear vacuoles in mouse LSECs (yellow), while KCs showed intense green staining in differently sized perinuclear vacuoles (**C**). Continuous phage uptake was also evident in human LSEC-enriched sinusoidal cultures, with K1F^gfp/488^ accumulating in FSA^647^-loaded compartments (**E**). Scale bar = 20 µm in all images.

Formaldehyde-treated serum albumin (FSA) is a scavenger receptor ligand (26, 27) and shown to be an excellent functional marker of the endo-/lysosomal compartment in rat LSECs (24). Results presented in Fig. 4; Fig. S2 confirm the localization of FSA in the endo-/lysosomal compartment also in human and mouse LSECs. Alexa Fluor 647-labeled FSA (FSA^647^) was observed to gather in early endosome antigen 1 (EEA1)-positive vacuoles in early phases of cell challenge (Fig. 4A; Fig. S2). At later timepoints, vacuoles loaded with FSA^647^ stained positive for LAMP-1 or were labeled by LysoTracker (Fig. 4B and C). Figure 5 demonstrates that internalized phages colocalize with the functional endocytic marker FSA in both murine and human LSECs. Live cell imaging of mouse and human LSECs showed that internalized K1F^gfp/488^ could be detected already after 35 min of continuous phage challenge with both the number and intensity of positive structures increasing with time (Fig. 5A and D; Video S1 and S2). Colocalization of phages with FSA^647^-positive vacuoles appeared after 35 min and accumulated until the 95 min timepoint. As the FSA challenge was given as an initial pulse chase, while the phage challenge was continuous throughout the live cell imaging, internalized phages could also be seen in non-FSA-containing compartments. The uptake of K1F^gfp/488^ was uniform throughout the culture, confirming that the internalization of small, icosahedral-shaped phages is a general feature of LSECs. Uptake of K1F^gfp/488^ was also apparent in mouse KCs (Fig. 5B and C) and human CD68-positive liver-associated macrophages (Fig. 5E; Fig. S3). As previously observed (28), KCs did not rapidly internalize FSA^647^ like LSECs, and colocalization with phage-positive vacuoles could not be evaluated. After 10 and 24 h of continuous phage challenge, intense green fluorescence in both LSECs and KCs confirmed persistent internalization of phages (Fig. 5C and E). No fluorescence was detected in hepatic stellate cells after 95 min or 24 h of continuous phage challenge (Fig. S4A and C). Similarly, very little fluorescence was associated with the few hepatocytes that occasionally appeared as contaminants in the mouse non-parenchymal cell (NPC) cultures (Fig. S4B and D).

To evaluate the inactivation of phage K1F^gfp^ following endocytosis in LSECs, cultured primary mouse LSECs were challenged with 10^8^ PFU K1F^gfp^/10^6^ cells for 1 h before viable phage particles (PFU) as well as total phage DNA (GEq) were quantified (Fig. S5). Cells incubated at 4°C were used as control for phage attachment without subsequent internalization. The mean amount of viable K1F^gfp^ after 1 h of incubation at 37°C was 1,665 PFU/well (*n* = 2, SD = 719), 73% less than the detected amount of viable K1F^gfp^ attached after 1 h of incubation at 4°C [mean 6,438 PFU/well (*n* = 2, SD = 1,874)]. Accordingly, cell-associated GEq (mean 4.09 × 10^5^, *n* = 2, SD = 1.68 × 10^5^) detected after 1 h at 37°C showed no reduction compared to the attachment controls. Cell-free controls of phage K1F^gfp^ incubated for 1 h at 37°C showed no loss of viability (results not shown).

## DISCUSSION

Localization of phages in the liver and spleen following clearance from blood has been shown for several types of phages (9, 10, 29–31) and is likely due to a high content of macrophages in these organs. Internalization of phages has further been shown in macrophage cell cultures [(32–34), reviewed in reference (35)], but a clear association to cell types responsible for phage uptake in the liver and spleen following blood clearance *in vivo* has so far not been established. We have previously shown that bacteriophage T4 is endocytosed by primary rat LSECs in culture, and they are rapidly shuttled to the lysosomal compartment following uptake (24). Our present study investigated early-stage blood clearance of the medium-sized, icosahedral-shaped K1F (T7-like) phage, with clearance kinetics in line with previous investigations of phage T7 blood clearance (36). Here, we demonstrate the essential scavenger role of the liver in phage capture. In addition, our results are the first to identify phage distribution in the liver at the hepatocellular level, confirming the shared workload of LSECs and KCs in the clearance of phage K1F^gfp^ from the circulation.

The spleen is often concluded to be the organ with the highest uptake of phage based on results shown in PFU/gram tissue (9, 37). When comparing organ distribution in the early phase of K1F^gfp^ blood clearance in the present study, the spleen seems to be the most efficient organ when calculated per gram tissue in mice receiving the high dose. However, the spleen (average weight 0.075 g, ±0.003 g, *n* = 6) is much smaller than the liver (average weight 1.104 g, ±0.034 g, *n* = 6). When calculating K1F^gfp^ distribution/total organ, it becomes clear that the liver contributes much more to the blood clearance with a total of 4.2–30.7 times more K1F^gfp^/organ compared to the spleen depending on the dose. This conclusion is in line with the early investigations of phage T4 blood clearance performed by Inchley (10). The rapid blood clearance of K1F^gfp^ illustrates the exceptional capacity of the liver to eliminate foreign matter from the circulation. In total, 9.74 × 10^6^ phage particles were removed from the blood within the first 20 min in the animals receiving the lower dose, and a striking amount of 5.72 × 10^9^ K1F^gfp^ particles was eliminated within this short period following administration of the higher dose. With the high dose, the clearance kinetics clearly increased, even though about 30% of phage particles were left in the circulation after 20 min. This may possibly reflect a temporary saturation of the scavenger cell system leading to increased circulatory presence of the phages, a result that is supported by the different distribution per milligram tissue in the spleen and liver between mice receiving a lower and a higher dose. In-depth knowledge about how the administered dose is affected by liver scavenging is vital when estimating the efficiency of applied doses in the therapeutical use of intravenously administered phages. Of note, doses commonly applied in intravenously administered phage therapy (10^9^ PFU) (38) translate to a distributed concentration of 2 × 10^5^ phage particles/mL blood in an average 70 kg patient. The low dose, nearly eliminated within 20 min in the present mouse model, was 25-fold higher with 5 × 10^6^ phage particles/mL blood [in an average 25 g mouse (39)]. For phage therapy to be effective, it is vital that enough phages escape the initial elimination by the liver to encounter their bacterial host for further multiplication. Moreover, modification of phages to reduce scavenger cell blood clearance could have a large impact on phage therapy efficiency.

Based on the knowledge of the dual cell principle of waste clearance in the liver, with particles above 200 nm being phagocytosed in KCs, while LSECs are involved in the clearance of soluble waste and nanoparticles below 100–200 nm in diameter (size limit of clathrin-mediated endocytosis) (13), it is not surprising that both KCs and LSECs contribute to the blood clearance of K1F^gfp^. Calculated per cell, the efficiency of phage K1F^gfp^ uptake is significantly higher in KCs compared to LSECs. Estimations have been made of the cellular composition of rat liver with LSECs comprising 2.5 times more the NPCs than KCs (40). Assuming the relative number of cells per organ weight follows the same distribution in the mouse liver, KCs are the main scavengers of K1F^gfp^ phage particles from the circulation. When phage uptake is extrapolated to the cell yields per organ in our experiments (6–12 × 10^6^ CD146+ LSECs and 0.25–0.5 × 10^6^ F4/80 and/or CD11b + KCs), it could be calculated that the KC fraction was responsible for 80% of the total phage uptake, while the LSEC fraction, which contains 97.3%–98.4% stabilin-2 positive LSECs, was responsible for 20% of the uptake. Stabilin-2/scavenger receptor (SR)-H2 is highly expressed in LSECs and a specific LSEC marker in rodent and human liver (26, 41–43).

Internalization of phages in cells is a major process determining phage pharmacokinetics in mammals (4) and consists of several different modes of endocytosis (44). Uptake of the filamentous phage M13 was in an inhibitor study shown to be different according to the cell type investigated, but caveolin-mediated endocytosis was the main mechanism in human dermal microvascular endothelial cells (45). Similarly, the internalization of filamentous phage Pf4 (temperate *Pseudomonas aeruginosa* phage) is mediated by receptor- and clathrin-mediated endocytosis in murine bone marrow-derived dendritic cells (46). Internalization mechanisms of icosahedral or head-tail-shaped phages are poorly investigated, but endocytosis of small- to medium-sized phages by clathrin- or caveolin-mediated endocytosis (<200 nm) is plausible.

KCs internalize foreign matter by either phagocytosis, macropinocytosis, or clathrin-mediated endocytosis depending on the size of the particle (47), while LSEC is a unique member of the endothelial cell family, performing extremely efficient clathrin-mediated endocytosis of an array of soluble ligands (27). This is in contrast to caveolin-mediated endocytosis, which is characteristic of many other types of endothelial cells (14). Endocytic scavenging in LSECs is receptor-mediated, whereas non-receptor-dependent endocytosis such as macropinocytosis is not a central feature in these cells (48). The scavenger receptor ligand FSA binds to stabilin-2 in LSECs and is shown to be taken up by clathrin-mediated endocytosis (26, 49). When given in low concentrations (10 µg/mL) for a short period (15 min), only LSECs are capable of effective internalization (27), while KCs show only low uptake, making this compound an efficient substrate to identify LSECs and label their endocytic compartments (24). The fact that internalized K1F^gfp/488^ colocalized with FSA-containing vacuoles already after 35 min in both mouse and human LSECs is a strong indication that clathrin-mediated endocytosis is the mode of uptake in these cells.

Blood clearance and uptake of several mammalian viruses in LSECs have been reported (16, 17, 22), but receptors responsible for binding and internalization are not yet identified. Srivastava et al. (2004) investigated the contribution of different immune cells to phage inactivation, concluding that a lack of B-cells increased the retention and survival of phage T7 in the circulation of female C57BL/10-Igh-6^tm1Cgn^ (B-cell deficient) mice (36). Furthermore, IgM and IgG depletion have been shown to prolong phage survival in human serum (50). The results from our *in vitro* assays confirm increased uptake of phages opsonized with plasma proteins in both LSECs and KCs. LSECs express the inhibitory, low-affinity endocytosis receptor FcɣRIIb2, which mediates efficient uptake of small, soluble immune complexes (51, 52), while KCs express several receptors capable of recognizing the Fc-portion of immunoglobulins (47, 53, 54). As natural antibodies against phages are found in both humans and animals (55, 56), both LSECs and KCs, being equipped with these Fc-receptors, can efficiently bind and internalize phage particles opsonized by natural antibodies. Formation of phage aggregates following antibody binding would favor phagocytosis by KCs, with the immune aggregates being recognized by phagocytic IgG Fc and C3b receptors. Acquired immunity and the development of neutralizing antibodies in patients receiving long-term phage treatment (57) may further contribute to increased clearance.

Lipopolysaccharide (LPS) derived from the bacterial host has been shown to adsorb to the surface of the filamentous Ff phages despite extensive purification (58). As LPS is effectively cleared from the bloodstream by the combined action of LSECs and KCs (59), such opsonization could enhance binding to scavenger receptors and cannot be completely ruled out in the present study. However, as the endotoxin level in our phage stock solution was low (8 EU/10^11^ PFU), this seems less likely to have a high impact on the observed results.

Phage K1F has been shown to be able to reach intracellular host bacteria (*Escherichia coli* K1) in human urinary bladder epithelial cells and cerebral microvascular endothelial cells (60, 61). Phage K1F was in these studies found to colocalize with phagosomal and lysosomal markers (RAB7, cathepsin-L, and LC3B), confirming their presence in intracellular vacuoles both with or without the presence of intracellular bacterial host *E. coli* EV36-RFP. Quantification of intracellular viable phages was not performed, but phage K1F was assumed to be degraded by LC3B-assisted phagocytosis. Intracellular survival of phage T4 was investigated in epithelial (A549), endothelial (HUVEC), and fibroblast (BJ) cell lines, with no significant differences between active and non-active intracellular phage particles after 18 h of continuous phage challenge (5). In contrast to these cell lines, LSECs have an extraordinarily high endocytic capacity given that internalized T4 phage accumulates in late endosomes/lysosomal compartments already at 25 min post challenge (24). Our results corroborate these findings with K1F^gfp/488^-containing vacuoles clearly colocalizing with FSA-loaded vacuoles within the first hour of continuous phage challenge in both mouse and human LSECs. Transportation along the endosomal route to lysosomes was apparent at later stages of the phage challenge, and reduced viability of internalized K1F^gfp^ confirmed inactivation of endocytosed phage particles.

In conclusion, our work shows the instrumental role of the liver in early blood clearance of the T7-like phage K1F^gfp^. Hepatocellular investigations confirmed that the elimination is performed by sinusoidal scavenger cells; the KCs and the LSECs, in collaboration. In lower doses, more than 95% of the phage particles were cleared from blood already within 20 min following injection, a process almost completely performed by the liver. In higher doses, the contribution from the spleen may reflect temporary saturation of the liver scavenging system. Opsonization of phage particles with plasma proteins contributed to increased cellular uptake, and internalized phages were shown to be transported along the endosomal apparatus to late endosomes/lysosomes, providing pieces of much-needed knowledge on the cellular mechanisms contributing to the rapid blood clearance and phage fate in liver sinusoidal cells. Future research should encompass early blood clearance of clinically applied phages with their bacterial hosts present to advance our understanding of efficient dosing in intravenously administered phage therapy, as well as the identification of phage variants with reduced affinity for liver sinusoidal cell scavenging.

## MATERIALS AND METHODS

### Animal model

C57BL/6JRj male mice (Janvier Labs, France) were group housed (3 mice/cage) in cages with aspen bedding (Scanbur, Norway), nesting material, houses, and aspen bricks (all provided by Datesand Ltd, Manchester, UK) as environmental enrichment. Fresh water and a standardized mouse diet were provided *ad libitum*. The mice were kept under controlled conditions (21℃ ± 1℃, relative humidity 55% ± 10%, and 12 h light/dark cycle) at the Unit of Comparative Medicine (AKM), the Animal Research Facility at UiT – The Arctic University of Norway. The animal study protocol was approved by the Norwegian Animal Food Safety Authority (permission no. 23052). The experimental protocol for primary liver cell isolation was approved by the competent institutional authority at UiT – The Arctic University of Norway, licensed by the National Animal Research Authority at the Norwegian Food Safety Authority (approval IDs: UiT 20/21 and 09/22). All experiments were performed in compliance with the European Convention for the Protection of Vertebrate Animals used for Experimental and Other Scientific Purposes and the Norwegian Animal Welfare Act.

### Human liver tissue and isolation of sinusoidal cells

Liver tissue was obtained from donors undergoing hepatic resection at the University Hospital of North Norway, Tromsø, Norway. Cells were isolated from tissue pieces with no macroscopic visible pathology. Patients included in the study gave informed consent, and ethical approval of the study was granted by the Norwegian Research Ethics Committee (approval reference number 7065). Perfusion of human liver tissue and cell isolation was performed according to reference (62), with minor modifications described in reference (63).

### Bacteriophage quantification and virion integrity assessments

Phage K1Fg10b::gfp (K1F^gfp^) stock solutions were assessed for virus titer and infectivity by qPCR and PFU determination, respectively, and phage virion integrity was verified by transmission electron microscopy (TEM; Fig. S1). A K1F^gfp^ stock solution with a concentration of 1.25 × 10^11^ GEq/mL, corresponding to 1 × 10^11^ PFU/mL, was used in all experiments. The concentration of endotoxin was 8 EU/mL. See supplementary information for bacteriophage propagation, endotoxin quantification, PFU, and TEM methods.

### Quantitative real-time PCR

An assay for quantification of GEq of phage K1Fg10b::gfp (K1F^gfp^) based on the sequence of the GFP plasmid (60) was developed. First, DNA was isolated from 20 µL of each batch of virus diluted in 180 µL phosphate-buffered saline (PBS) by using a QIAamp DNA Mini Kit (QIAGEN, cat# 51304) following the manufacturer’s recommendations. The final elution volume was 100 µL. DNA purity and concentration were assessed with a Nanodrop 2000 Spectrophotometer (Thermofisher Scientific, USA) before further use. Primers and probes specific to phage K1F^gfp^ were designed against the GFP-tag of the phage (Table S2). The PCR reaction was run using a QuantStudio 5 real-time PCR system (Applied Biosciences), with TaqMan Fast Advanced Master Mix (Thermofisher, cat# 4444556), using the provider’s protocol (95°C for 20 s followed by 40 cycles of 1 s at 95°C and 20 s at 60°C). Each reaction’s final volume was 20 µL, including 2 µL of DNA, and the final concentration of primers and probe was 500 and 100 nM, respectively. For the standard curves, gBlocks were designed and diluted for a stock concentration of 10^9^ GEq/μL, and 10-fold serial dilutions ranging from 10^8^ to 10^1^ GEq/μL were included in triplicates in each plate (Table S3). DNA isolated from K1F^gfp^ was used as an internal positive control, while nuclease-free water was used as NTC. Serial dilutions for the standard curve were stored at −20°C until further use and discarded after a maximum of five freeze-thaw cycles.

### *In vivo* viral distribution

The experimental setup is described in Fig. 1. In detail, mice (*n* = 3/dose) kept in surgical anesthesia received either 1 × 10^7^ (low dose) or 6.45 × 10^9^ (high dose) GEq of phage K1F^gfp^ via the tail vein (ratio GEq:PFU = 1.25:1 in stock solution, the low dose contained 0.6 × 10^−3^ EU, and the high dose contained 0.4 EU of endotoxin). The anesthesia protocol consisted of gas inhalation of isoflurane with an induction dose of 4% and a maintenance dose of 2% at a flow rate of 0.6 L/min. In addition, 0.05 mg/kg buprenorphine was administered s.c. 30 min before phage injection, then the tip of the tail was cut, and blood samples were collected at 1-, 3-, 5-, 7-, 10-, and 20-min post injection. Samples of up to 50 µL of blood were collected in EDTA-coated Microvette 100 K3E tubes (Sarstedt AG & Co, cat# 20.1278.100) and kept at −20°C for at least 24 h and until further processing. The animals were euthanized by cervical dislocation immediately after the last blood sample, and selected organs were harvested for viral quantification. Subsamples of liver right lobe (R liver), liver left lobe (L liver), spleen, kidneys, heart, lung, and brain were finely cut in pieces, placed in 2.0 mL DNA LoBind tubes (Eppendorf, cat# 0030108078) and immediately frozen at −20°C for downstream DNA isolation.

### Blood and tissue DNA isolation

DNA was isolated from blood samples using GenElute Single Spin Blood DNA kits (Sigma Aldrich, cat# EC100-250RXN) and from tissue samples using GenElute Single Spin Tissue DNA kits (Sigma Aldrich, cat# EC300-250RXN) following the manufacturer’s instructions. After thawing, tissue samples were weighed, and samples of 10–25 mg, depending on the organ, were placed in 2.0 mL DNA LoBind Tubes for further processing. DNA (60–90 μL) samples were stored at –20°C until further analysis as described in the qPCR section.

### Hepatocellular distribution of phage K1F^gfp^

Mouse primary liver sinusoidal cells (LSECs and KCs) were isolated as described (64). In brief, mice (*n* = 3) were anesthetized with isoflurane, injected with 1 × 10^8^ GEq of K1F^gfp^ via the tail vein (ratio GEq:PFU = 1.25:1 in the stock solution, and the total dose of 1 × 10^8^ phage contained 0.6 × 10^−2^ EU of endotoxin) and euthanized by cervical dislocation after 5 min. Blood samples were taken 1–3 min after injection from the saphenous vein and immediately after euthanasia from the inferior vena cava to verify the correct delivery of phages and virus decrease in the circulation. The liver was perfused as reported by reference (64) with 0.024 mg/mL of Liberase (Roche, cat# 5401127001) in perfusion buffer with 4.76 mM CaCl_2_. In total, 30–40 mL of Liberase buffer was passed through the liver before digestion was completed. Parenchymal cells were removed from the cell suspension by a series of centrifugations (50 × *g* for 2 min at 4°C). NPCs were pelleted by a 10-min centrifugation at 300 × *g* before proceeding with their purification. Mouse LSECs were isolated by positive selection using CD146-conjugated magnetic microbeads (MACS, Miltenyi Biotec, cat# 130–092-007), while KCs were isolated by positive selection using a combination of F4/80-conjugated beads (MACS, Miltenyi Biotec, cat# 130–110-443) and CD11b-conjugated beads (MACS, Miltenyi Biotec, cat# 130–049-601). Of the total NPCs isolated per liver, 1/3 of the sample was used for the purification of LSECs, while 2/3 of the sample was used for the purification of KCs. Immediately after the purification steps, most cells were frozen in 200 µL PBS to proceed with DNA isolation, while a subsample of the cell suspension was plated for purity assessment by immunostaining for cellular markers.

### *In vitro* phage challenge

Approximately, 2.5 × 10^5^ LSECs or 1.75 × 10^5^ KCs, both freshly isolated, were seeded onto fibronectin (0.2 mg/mL)-coated 48-well tissue culture plates (Falcon, cat# 353078) in 250 µL serum-free RPMI-1640 (LSECs) or AIM-V (Gibco, cat# 12055–091; KCs). The culture plates were incubated for 30 min at 37°C in an atmosphere containing 5% O_2_ and 5% CO_2_ to allow the cells to adhere to the plate. The cells were gently washed and incubated in AIM-V with 0.1 µM dexamethasone (Fortecortin, cat# 266763) overnight. LSECs were then kept in serum-free RPMI-1640 with 100 IU penicillin/100 µg/mL streptomycin, and KCs were kept in AIM-V without dexamethasone for 1 h before being challenged with 0.5 × 10^8^ PFU of K1F^gfp^/well (in total 0.4 × 10^−2^ EU of endotoxin/well) in a total of 150 µL medium with or without 1% mouse plasma (Sigma-Aldrich, cat# P9275). Opsonized phages were incubated with mouse plasma for 30 min at RT prior to infective stock preparation. Control wells were incubated with phage stock for 5 min at RT before being washed five times with medium, refilled with medium, and harvested at the 1 h timepoint. Cell cultures were incubated for indicated times at 37°C to allow for internalization of K1F^gfp^. The phage suspension was removed, and cells were carefully washed five times with medium, detached by scraping, and collected in 250 µL cold PBS. Samples for qPCR were kept on ice during collection and stored at −20°C until further processing. Samples from the last wash were also collected to determine background levels of non-internalized/unbound phages. See supplementary material for intracellular survival of phage K1F^gfp^.

### *In vitro* live cell imaging

1.5 × 10^6^ freshly isolated LSECs or NPCs from LSEC-isolation flowthrough, enriched in KCs and hepatic stellate cells, were seeded in fibronectin-coated (0.2 mg/mL) 35 mm dishes (Ibidi μ-Dish, cat# 81156) in 500 µL AIM-V. The dishes were incubated for 30 min at 37°C in an atmosphere containing 5% O_2_ and 5% CO_2_ to allow the cells to adhere. The cells were gently washed and incubated in AIM-V with 0.1 µM dexamethasone overnight. To allow for visualization in primary cells with moderate background autofluorescence, phage K1F^gfp^ was labeled with Alexa Fluor 488 (Invitrogen, cat# A20181; K1F^gfp/488^) according to the manufacturer’s instructions. Cultures were pre-treated with FSA^647^ (10 µg/mL) for 15 min and given a chase of 30 min in medium alone. FSA is a scavenger receptor ligand that is taken up by endocytosis in LSECs and shown to be an excellent functional marker for the endocytic compartment of these cells (24). Visualization of lysosomes was performed using LysoTracker Red DND99 (Invitrogen, cat# L7528, 100 nM). Live cell imaging was performed in a Zeiss LSM800 confocal microscope, equipped with 405, 488, 561, and 649 diode lasers and an incubation chamber set to 37℃ and 5% CO_2_, using a 40× water immersion lens. Cells were imaged before the phage challenge and every 15 min after the addition of 2 × 10^9^ PFU K1F^gfp/488^ for a total of 95 min. Cultures continuously challenged with K1F^gfp/488^ were also imaged after 10 and 24 h.

### Statistical analyses

qPCR data were analyzed in the Design & Analysis Software version 2.6.0 (Thermo Fisher Scientific Inc.) and exported in .csv format for further analyses. Data were normalized, and average values and SDs were calculated with Microsoft Excel “=AVERAGE(array)” and “=STDEV(array)” functions. Mann-Whitney tests on *in vitro* data were performed using GraphPad Prism version Prism 9 for macOS, GraphPad Software, San Diego, California USA, www.graphpad.com.

## Data Availability

The data presented in this study are available in the article or in the supplementary material.

## SUPPLEMENTAL MATERIAL

Supplemental material msphere.00702-23-s0001.docx Supplemental methods, Figures S1 to S5, Tables S1 to S3.

Video S1 msphere.00702-23-s0002.mpeg Live cell imaging of mouse primary CD146+ LSECs.

Video S2 msphere.00702-23-s0003.mp4 Live cell imaging of human primary CD31/CD146+ enriched sinusoidal cells.

ASM does not own the copyrights to Supplemental Material that may be linked to, or accessed through, an article. The authors have granted ASM a non-exclusive, world-wide license to publish the Supplemental Material files. Please contact the corresponding author directly for reuse.
