# Supplementary material for: Liver sinusoidal cells eliminate blood-borne phage K1F
Source: mSphere. 2024 Feb 28;9(3):e00702-23. doi: 10.1128/msphere.00702-23 (PMC10964407; doi:10.1128/msphere.00702-23)
Supplement: Supplemental material — Supplemental methods, Figures S1 to S5, Tables S1 to S3. [file msphere.00702-23-s0001.docx]

Supplementary information

*K1F^gfp^ stock preparations*

Phage K1Fg10b::gfp (K1F^gfp^) were propagated in *Escherichia coli* EV36 according to previously published protocols (1). Following serial incubations of phage and host to obtain sufficient volume for high concentrations, the bacterial lysate was centrifuged at 4,000 x *g* for 15 min to remove cell debris before NaCl to a final concentration of 1 M was added. The lysate was incubated on ice for 1 h and centrifuged at 4,500 x *g* for 45 min. The supernatant was recovered, filtered through 0.2 μm Filtropur S filters (Sarstedt AG & Co, cat# 83.1826.001) and incubated with polyethylene glycol 800 (VWR, cat# 0159) at a final concentration of 10% w/v on ice at 4^o^C overnight. The solution was then centrifuged at 25,000 x *g* for 60 min at 4^o^C to precipitate phage particles and the resulting pellet was dissolved in SM-buffer 1 (1 M NaCl, 8 mM MgSO_4_·7H_2_O, 25 mM Tris-HCl). The phage suspension was purified using a CsCl gradient of three densities, 1.7 g/mL, 1.5 g/mL, and 1.4 g/mL, with CsCl added to the phage suspension to a final concentration of 1.3 g/mL and layered on top in 14 mL SW40Ti open-top thinwall polypropylene ultracentrifuge tubes (Beckman Coulter, cat# 331374). The gradients were centrifuged at 150,000 x *g* for 20 h at 4^o^C and the resulting band harvested by piercing the tube with a large gauge syringe needle. The purified phage suspension was thereafter dialyzed to remove CsCl. 20 K MWCO 3 mL Slide-A-Lyzer dialysis cassettes (Pierce, cat# 66003) were incubated in SM buffer 1 overnight at 4^o^C, before being transferred to SM buffer 2 (100 mM NaCl, 8 mM MgSO_4_·7H_2_O, 25 mM Tris-HCl) for two times 2 h at room temperature (RT). Purified phage suspensions were stored at 4^o^C. The ToxinSensor™ Chromogenic LAL Endotoxin Assay Kit (Genscript) was used to measure the amount of endotoxin (LPS) in the phage preparations and the analysis was performed by Jafral d.o.o. (Ljubljana, Slovenia).

*Plaque-forming unit (PFU) determination*

Serial dilutions of phage K1F^gfp^ stock in phosphate buffered saline (PBS) were prepared. Selected phage dilutions (100 μL) were incubated with 100 μL of liquid culture of mid-log *E. coli* EV36 cells (OD_600_ = 0.1) for 15 min at RT before being mixed with 3 mL warm (cooled to 44°C) overlay agar (lysogeny broth (LB), 0.7% agar) and transferred to LB agar plates at RT. Plates were left to solidify before being incubated at 37°C overnight. Samples for PFU determination were always prepared in duplicate plates. Plaque numbers were counted the following day and used to calculate phage titers in PFU/mL.

*Transmission electron microscopy*

Phage suspension was diluted 1:2 in 8% formaldehyde (FA) (Sigma-Aldrich, cat# 158127) in PHEM for a final concentration of 4% FA. The fixed viral suspension was then processed for negative staining and TEM in duplicates. In short, 400 mesh formvar coated copper grids were glow discharged at 10 mA for 10 s in an EC-52000K Ion cleaner (Brand). Grids were then placed on top of 5 μL drops of the viral suspension at RT for 5 min in a moist chamber and subsequently washed in ddH_2_O four times. Grids were placed on droplets containing 1% uranyl acetate (UA) in ddH2O at RT for 20 s before being carefully picked up with watchmaker forceps, removing excess UA with filter paper and air-dried for at least 10 min before storage. Stained grids were examined with a Jeol JEM 1010 transmission electron microscope (Jeol USA Inc., Peabody, MA, USA) connected to a Morada Camera system (Olympus Soft Imaging Solutions, Münster, Germany) (Supplementary figure 1).

*Purity assessment*

A subsample of the cells isolated in the hepatocellular distribution study were seeded onto fibronectin (0.2 mg/mL)-coated 8-well plates (Ibidi, μ-slide, cat# 80826) and incubated for 20-30 min at 37^o^C in an atmosphere containing 5% O_2_ and 5% CO_2_ to allow for adherence. Approximately 1x10^5^ cells purified by CD146 MACS, and 3x10^4^ cells purified by F4/80/CD11b MACS were seeded per well, respectively. The cultures were gently washed and kept in AIM-V medium for 1 h, then fixed at RT for 30 min in 4% FA (Sigma-Aldrich, cat# 158127) in PHEM buffer, pH 7. Cells were permeabilized with 0.2% Triton-X100 (Sigma-Aldrich, cat# X100) in phosphate-buffered saline (PBS) (VWR, cat# E404) for 2 min at RT, and blocked for 30 min in 1% bovine serum albumin (BSA) (AppliChem, cat# A1391) and 3% donkey serum (Sigma-Aldrich, cat# D9663) in PHEM buffer. Cells were stained for stabilin-2 as an LSEC-specific marker (2-5) (rat anti-mouse stabilin-2, MBL Life Science, cat# D317-3, 10 μg/mL) and VSIG4 (CRIg) as a KC marker (6) (goat anti-mouse VSIG4, Bio-techne Ltd., cat# AF4674, 5 μg/mL). Secondary antibodies were donkey anti-rat IgG H&L, Dylight 488 (Invitrogen, cat# SA5 10026, 10 µg/mL) and donkey anti-goat IgG Alexa 647 (Abcam, cat# ab150135, 10 µg/mL). Blocked cells were incubated for 60 min at RT with the primary antibody and 30 min with the secondary antibody, washing trice with PHEM buffer following each incubation step. Nuclei were counterstained with 4’,6-diamidino-2-phenylindole (DAPI) (Sigma-Aldrich, cat# D8417). Imaging was performed using the EVOS M5000 Imaging System (Invitrogen) equipped with LED-light cubes for DAPI, GFP, RFP, and Cy5. Ten images were captured at random per culture, using the 20x objective, including at least 2479 CD146+ and 1281 F4/80-CD11b+ cells per analysis. Cells were counted with Cellprofiler ([www.cellprofiler.org](http://www.cellprofiler.org)) (7) and the Cell counter plugin from Fiji was used to manually count the contaminant cells in each fraction (8). In the CD146+ cell fraction (LSECs), VSIG4 positive cells were contaminants and in the F4/80-CD11b+ cells (KCs) the stabilin-2 positive cells were contaminants. The purity of each fraction was calculated as the percentage of total cells minus contaminants. Total yield of each cell type/liver is extrapolated based on weight of the fraction of liver tissue included in the cell isolation procedure.

*Immunostaining*

Cultured sinusoidal liver cells were fixed for 30 min at RT using 4% buffered FA and permeabilized with 0.2% Triton X-100 in PBS for 4 min. Unspecific binding was blocked using 1% BSA and 3% donkey serum in PHEM buffer for 30 min. Immune labelling was performed using goat anti-human mannose receptor (hMR, CD206) antibody (R&D systems, cat# AF2534, 4 µg/mL), mouse anti-human CD68/SR-D1 antibody (Novus Biologicals, cat# NB100-683SS, 10 µg/mL), rabbit anti-human early endosome antigen 1 (EEA1) antibody (Santa Cruz, cat# sc33585, 4 µg/mL) and mouse anti-human LAMP-1 antibody (Developmental studies hybridoma bank, cat# H4A3, 0.3 µg/mL). Secondary antibodies were Alexa Fluor (AF) 488 donkey anti-mouse IgG, AF488 donkey anti-rabbit IgG, AF546 donkey anti-rabbit IgG, AF555 donkey anti-mouse IgG and AF568 donkey anti-goat IgG (Invitrogen, 4 µg/mL).

*Intracellular survival of phage K1F^gfp^ in mouse LSECs*

9 x 10^5^ freshly isolated mouse LSECs were seeded in fibronectin (0.2 mg/mL) coated wells of 12-well cell culture plates (Falcon, cat# 353043) with 1 mL serum-free AIM-V Medium (Gibco, cat# 12055-091). The culture plates were incubated for 30 min at 37℃ in an atmosphere containing 5% O_2_ and 5% CO_2_ to allow the cells to adhere to the plate, before being gently washed and incubated in AIM-V with 0.1 μM dexamethasone (Fortecortin, cat# 266763) overnight. The cells were kept in serum-free RPMI-1640, 100 IU penicillin/100 ug/mL streptomycin for 1 h before challenged with 10^8^ PFU of K1F^gfp^/well in a total of 500 μL RPMI. Cell culture plates were incubated for 1 h at 37℃ to allow for internalization or at 4℃ to control for phage cellular attachment without internalization. After incubation, wells were washed 3 times with RPMI to remove non-adherent phages before cells were scraped and collected in 500 μL cold PBS. Paired analyses for PFU and GEq were performed from the same wells. Subsamples for qPCR were stored at -20℃ until further processing, while subsamples for PFU were kept on ice and proceeded with immediately. Cells were here pelleted and resuspended in 200 μL cold 0.2% Triton X-100 in PBS to release intracellular phages. To ensure complete cell lysis, samples were passed 3x though a 29G syringe needle. Samples were then serial diluted in PBS to prepare for PFU determination. The experiment was repeated twice, each with cells isolated from one mouse seeded in parallel wells/treatment.

**Fig S1**


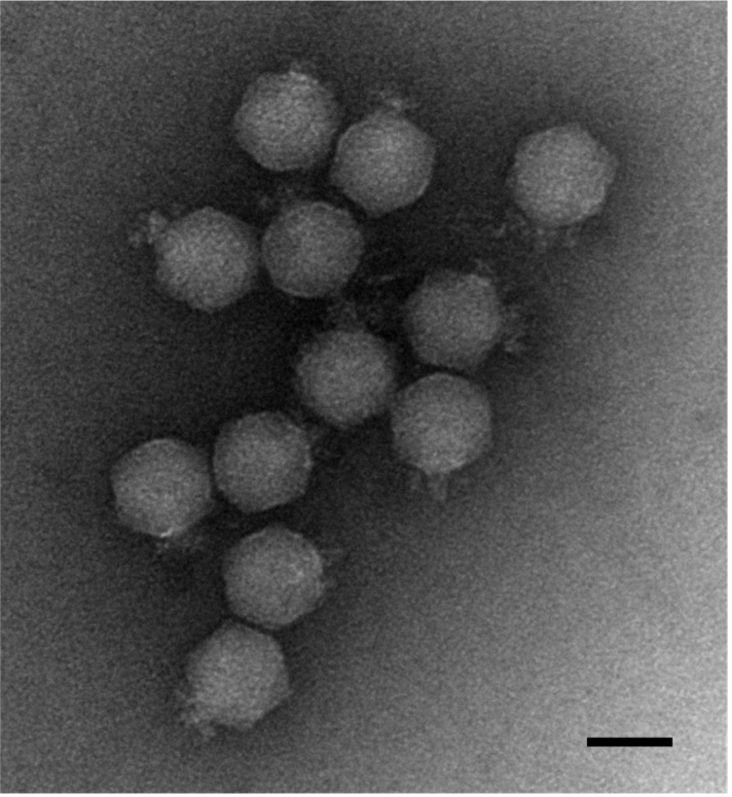


Supplementary Figure 1**.** *Transmission electron micrographs of the purified viral suspension showing the intact morphology of bacteriophage K1F^gfp^*. The phage particles have an icosahedral capsid and a short, non-contractile tail composed of six tail fibers. Scale bar represents 50 nm.

**Fig S2**


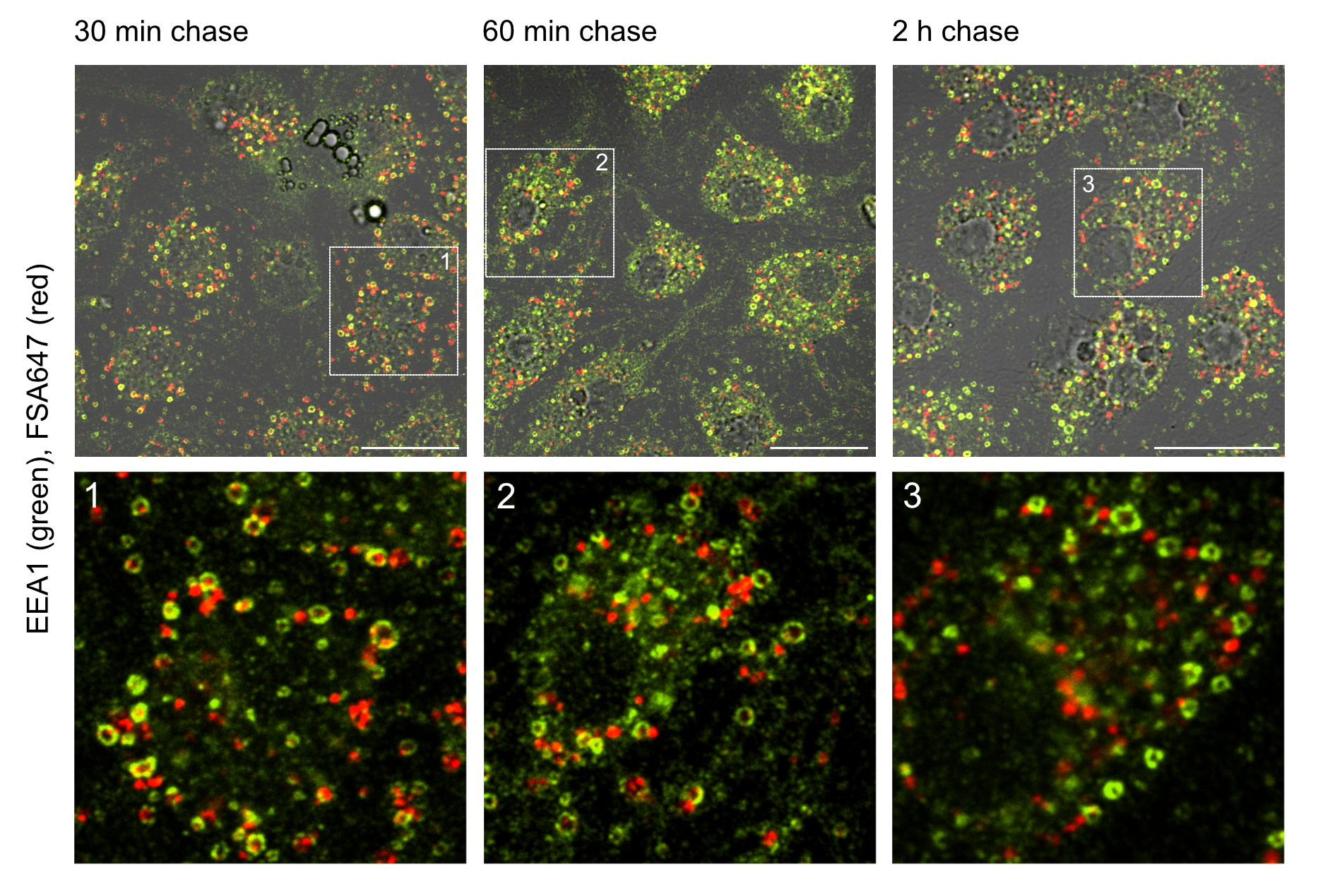


Supplementary figure 2. *Colocalization of scavenger receptor ligand FSA with early endosomes in mouse primary LSECs.* Mouse CD146+ LSECs were challenged with 10 μg/mL Alexa 647-labelled formaldehyde-treated serum albumin (FSA^647^, red) for 15 min and pulse-chased for indicated times. After 30 min, FSA^647^ (red) was localized in EEA1-positive vacuoles (green). At 1 h, the distribution of FSA^647^ in EEA1-positive vacuoles is less obvious, although present in some areas. After a 2 h chase period, FSA^647^ had been shuttled to a downstream compartment that does not express EEA-1. Scale bar = 20 μm.

**Fig S3**

*
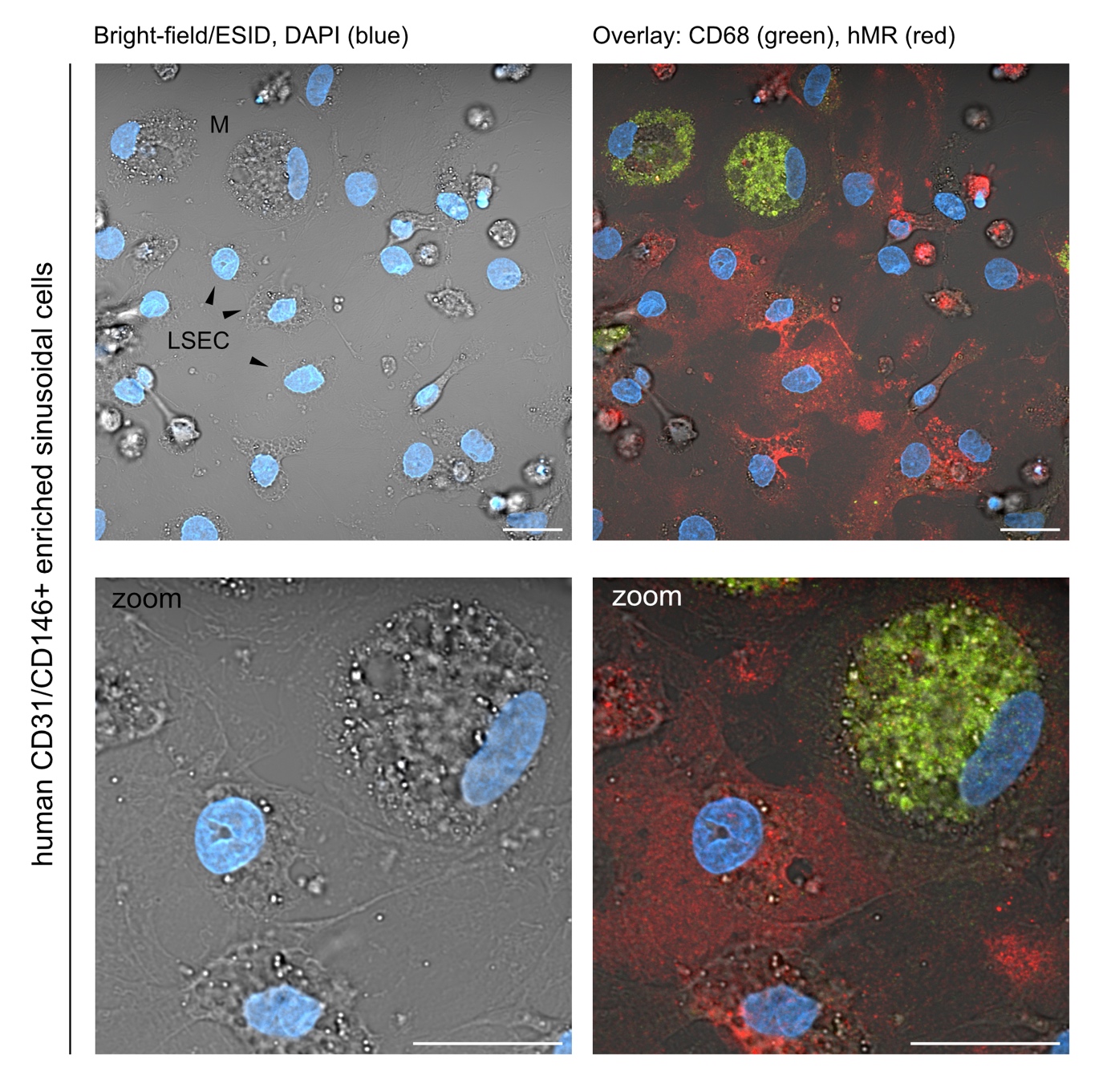
*

Supplementary Figure 3. *Cellular markers in human CD31/CD146+ enriched sinusoidal cells.*

Immunostaining of human CD31/CD146+ enriched sinusoidal cell cultures shows that cells with macrophage-like morphology express CD68 (green) and hMR (red), while cells with LSEC morphology are CD68-negative and hMR-positive. Scale bar = 20 μm.

**Fig S4**


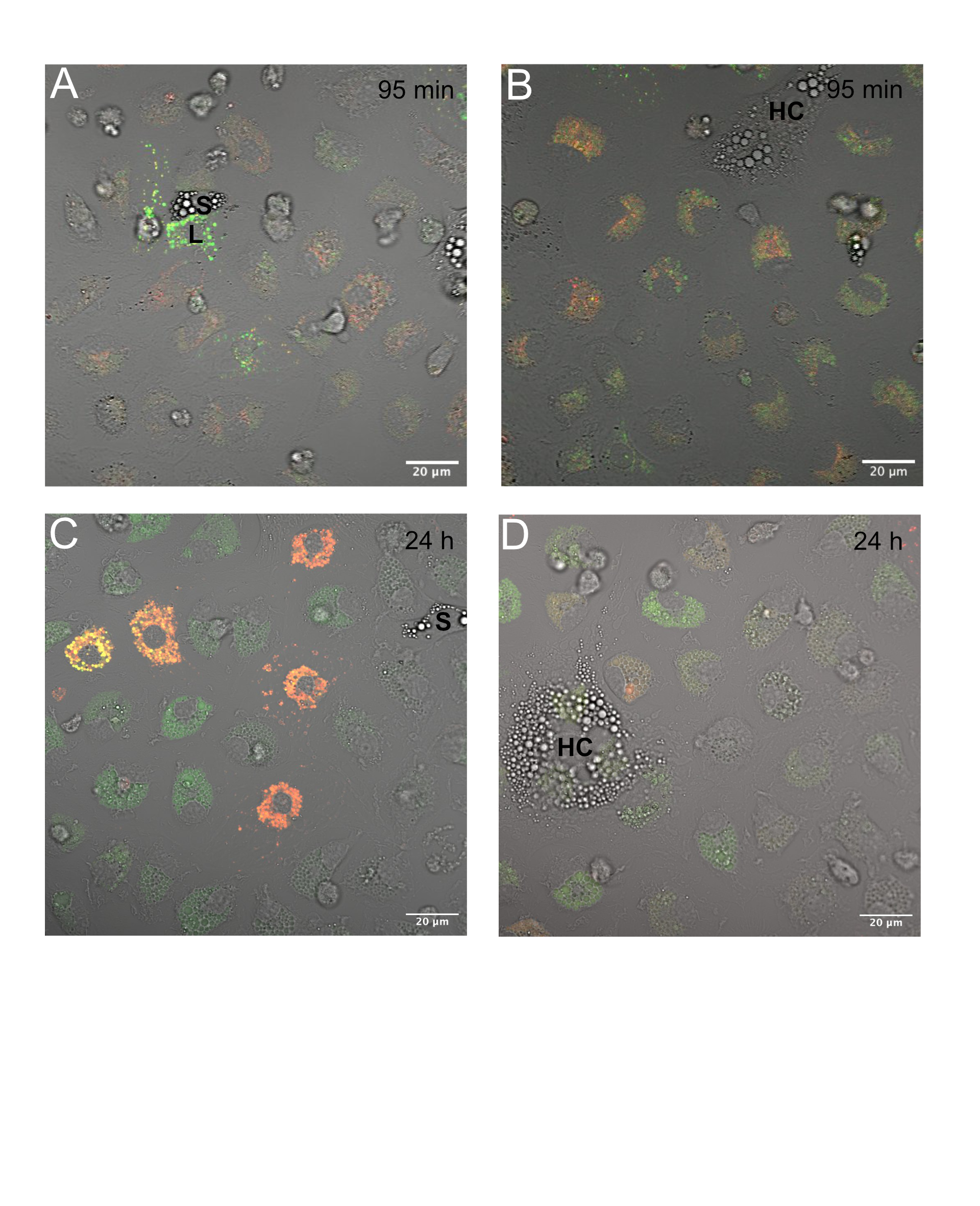


Supplementary Figure 4. *Live cell imaging and lack of phage uptake in hepatic stellate cells and hepatocytes.* Continuous challenge of mouse non-parenchymal cell (NPC) LSEC isolation flowthrough, enriched in KCs and hepatic stellate cells, in culture with 2x10^9^ PFU per 1.5x10^6^ cells confirmed uptake of K1F^gfp/488^ (green) in LSECs (L) and KCs, but not in hepatic stellate cells (S) (panel A and C). No fluorescence was associated with the few contaminating hepatocytes (HC) after 95 min (panel B) and only very little fluorescence could be seen after 24 h (panel D). LSECs in the cultures were identified by a pre-treatment with Alexa Fluor™ 647-labelled formaldehyde-treated serum albumin (FSA^647^) (10 μg/mL) for 15 min and given a chase of 30 min in medium alone before the phage challenge. No uptake of the scavenger receptor ligand FSA^647^ (red) was observed in either stellate cells or hepatocytes at any of the timepoints, and very little was associated with the KCs. Images were taken of live cells using a Zeiss LSM800 confocal microscope equipped with an incubation chamber set to 37℃ and 5% CO_2_.

**Fig S5**


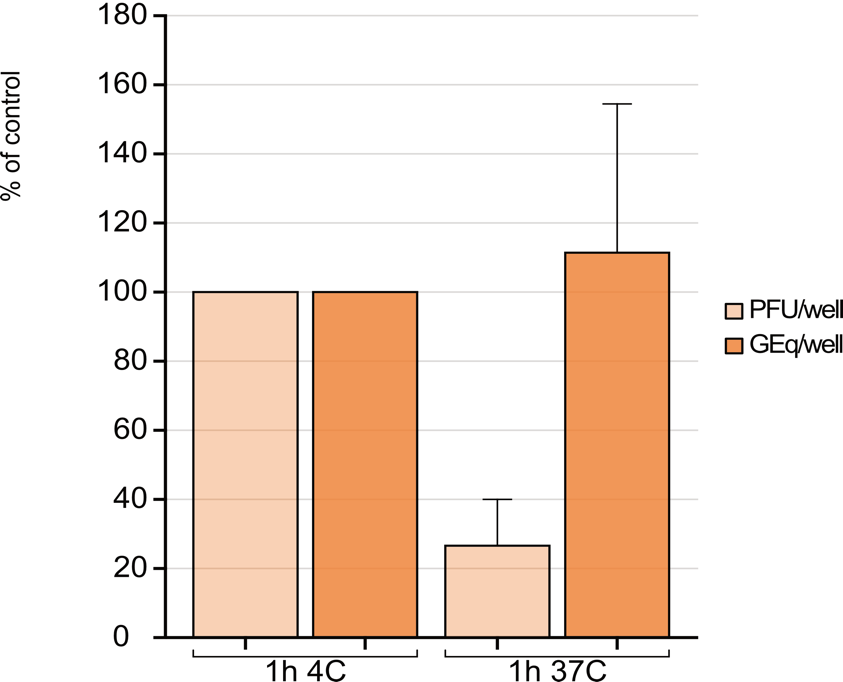


Supplementary figure 5. *Inactivation of phage K1F^gfp^ in primary mouse LSECs* *in vitro*. Cells were challenged with 10^8^ PFU K1F^gfp^/well for 1 h at either 4℃ (control) or 37℃. Viable cell-associated phages were determined by plaque assay (PFU/well) and total amount of cell-associated phage numbers were determined by qPCR (GEq/well). Paired analyses for PFU and GEq were performed from the same wells. Bars show the mean of 4 wells run in two separate assays and is given in percent compared to control. Error bars represent standard deviation.

Supplementary table 1. *Quantification of K1F^gfp^ in selected tissues.* Average of total amounts of viral GEq/mg tissue weight and average of total organ weights of the C57BL/6JRj mice injected with either a lower dose (10^7^) or a higher dose (6.45x10^9^) of K1F^gfp^ bacteriophage.

| Lower Dose (n=3) | | | | | Higher Dose (n=3) | | | | |
| --- | --- | --- | --- | --- | --- | --- | --- | --- | --- |
| Organ | GEq/mg tissue | SEM | Organ weight (g) | SEM | Organ | GEq/mg tissue | SEM | Organ weight (g) | SEM |
| Lung | 1.62x10^2^ | 9.35x10^1^ | 0.146 | 0.001 | Lung | 1.73x10^3^ | 3.20x10^2^ | 0.195 | 0.021 |
| Liver | 8.80x10^2^ | 3.03x10^2^ | 1.237 | 0.069 | Liver | 2.36x10^4^ | 6.62x10^3^ | 0.970 | 0.105 |
| Spleen | 5.74x10^2^ | 2.74x10^2^ | 0.062 | 0.001 | Spleen | 6.08x10^4^ | 2.28x10^4^ | 0.089 | 0.008 |
| Heart | 3.63x10^1^ | 1.06x10^1^ | 0.133 | 0.002 | Heart | 2.22x10^2^ | 1.32x10^2^ | 0.128 | 0.010 |
| Kidney | 1.97x10^1^ | 1.42x10^1^ | 0.312 | 0.013 | Kidney | 1.72x10^3^ | 1.47x10^3^ | 0.414 | 0.018 |
| Brain | 0 | 0 | 0.414 | 0.008 | Brain | 0 | 0 | 0.430 | 0.006 |

Supplementary Table 2. *Primers and probes.* Information about primers and probes used. The resulting amplicon has a length of 119 base pairs.

| **Name** | **Sequence (5´-3´)** | **Size** | **Final conc.** |
| --- | --- | --- | --- |
| GFP-tag Forward | GGTGAAGGTGACGCAACTAA | 20 | 500 nM |
| GFP-tag Reverse | GCAAAGCACTGAACACCATAAG | 22 | 500 nM |
| GFP-tag probe | /56-FAM/TACCTTGGC/ZEN/CGACTCTGGTAACGA/3IABkFQ/ | 24 | 100 nM |

Supplementary Table 3. *Quantitative polymerase chain reaction assay validation.* The qPCR assays performance was determined performing calibration curves with gBlock Gene fragments (Integrated DNA Technologies, Inc., USA), specifically designed for the target sequences. The linear range to perform calibration curves was from 10 – 10^6^ copies per well. Slope, y-intercept, R2 and PCR efficiency from the calibration curves are shown.

| **Target** | **Range (copies/ µl)** | **Slope** | **Y-Intercept** | **R2** | **Efficiency (%)** |
| --- | --- | --- | --- | --- | --- |
| GFPtag | 10^6^ - 10^1^ | -3.415 | 36.791 | 0.998 | 96.241 |
| GFPtag | 10^6^ - 10^1^ | -3.465 | 38.617 | 0.998 | 94.356 |

Supplementary Video 1. *Live cell imaging of mouse LSECs.* Mouse primary CD146+ LSECs were challenged with 2x10^9^ PFU of Alexa Fluor™ 488-labelled K1F^gfp^ (K1F^gfp/488^, green fluorescence). Cultures were pre-treated with Alexa Fluor™ 647-labelled formaldehyde-treated serum albumin (FSA^647^, red fluorescence) (10 μg/mL) for 15 min and given a chase of 30 min in medium alone to label the endocytic compartment of these cells. Images were captured every 15 min for a total of 90 min after addition of phage. Scale bar = 20 μm.

Supplementary Video 2. *Live cell imaging of human liver sinusoidal endothelial-enriched cells.* Human primary CD31/CD146+ enriched sinusoidal cells were challenged with 2x10^9^ PFU of Alexa Fluor™ 488-labelled K1F^gfp^ (K1F^gfp/488^, green fluorescence). Cultures were pre-treated with Alexa Fluor™ 647-labelled formaldehyde-treated serum albumin (FSA^647^, red fluorescence) (10 μg/mL) for 15 min and given a chase of 30 min in medium alone to label the endocytic compartment of LSECs. Images were captured every 15 min for a total of 90 min after addition of phage. Scale bar = 20 μm.

**References**

1. Møller-Olsen C, Ho SFS, Shukla RD, Feher T, Sagona AP. 2018. Engineered K1F bacteriophages kill intracellular Escherichia coli K1 in human epithelial cells. Sci Rep 8:1-18.

2. Bhandari S, Li R, Simón-Santamaría J, McCourt P, Johansen SD, Smedsrød B, Martinez-Zubiaurre I, Sørensen KK. 2020. Transcriptome and proteome profiling reveal complementary scavenger and immune features of rat liver sinusoidal endothelial cells and liver macrophages. BMC Mol Cell Biol 21:1-25.

3. Falkowski M, Schledzewski K, Hansen B, Goerdt S. 2003. Expression of stabilin-2, a novel fasciclin-like hyaluronan receptor protein, in murine sinusoidal endothelia, avascular tissues, and at solid/liquid interfaces. Histochem Cell Biol 120:361-369.

4. McCourt P, Smedsrød B, Melkko J, Johansson S. 1999. Characterization of a hyaluronan receptor on rat sinusoidal liver endothelial cells and its functional relationship to scavenger receptors. Hepatology 30:1276-1286.

5. Politz O, Gratchev A, McCourt PA, Schledzewski K, Guillot P, Johansson S, Svineng G, Franke P, Kannicht C, Kzhyshkowska J. 2002. Stabilin-1 and− 2 constitute a novel family of fasciclin-like hyaluronan receptor homologues. Biochem J 362:155-164.

6. Helmy KY, Katschke KJ, Gorgani NN, Kljavin NM, Elliott JM, Diehl L, Scales SJ, Ghilardi N, van Lookeren Campagne M. 2006. CRIg: a macrophage complement receptor required for phagocytosis of circulating pathogens. Cell 124:915-927.

7. Stirling DR, Swain-Bowden MJ, Lucas AM, Carpenter AE, Cimini BA, Goodman A. 2021. CellProfiler 4: improvements in speed, utility and usability. BMC Bioinformatics 22:1-11.

8. Schindelin J, Arganda-Carreras I, Frise E, Kaynig V, Longair M, Pietzsch T, Preibisch S, Rueden C, Saalfeld S, Schmid B. 2012. Fiji: an open-source platform for biological-image analysis. Nat Methods 9:676-682.
